# Identification of Representative Genes of the Central Nervous System of the Locust, *locusta migratoria manilensis* by Deep Sequencing

**DOI:** 10.1673/031.012.8601

**Published:** 2012-07-24

**Authors:** Zhengyi Zhang, Zhi-Yu Peng, Kang Yi, Yanbing Cheng, Yuxian Xia

**Affiliations:** ^1^ Genetic Engineering Research Center, School of Bioengineering, Chongqing Engineering Research Center for Fungal Insecticide, The Key Laboratory of Gene Function and Expression Regulation, Chongqing University Chongqing 400030, China..; ^2^ Beijing Genomics Institute-Shenzhen, Shenzhen 518083, China.

**Keywords:** database, transcriptome, unigene

## Abstract

The shortage of available genomic and transcriptomic data hampers the molecular study on the migratory locust, *Locusta migratoria manilensis* (L.) (Orthoptera: Acrididae) central nervous system (CNS). In this study, locust CNS RNA was sequenced by deep sequencing. 41,179 unigenes were obtained with an average length of 570 bp, and 5,519 unigenes were longer than 1,000 bp. Compared with an EST database of another locust species *Schistocerca gregaria* Forsskåi, 9,069 unigenes were found conserved, while 32,110 unigenes were differentially expressed. A total of 15,895 unigenes were identified, including 644 nervous system relevant unigenes. Among the 25,284 unknown unigenes, 9,482 were found to be specific to the CNS by filtering out the previous ESTs acquired from locust organs without CNS's. The locust CNS showed the most matches (18%) with *Tribolium castaneum* (Herbst) (Coleoptera: Tenebrionidae) sequences. Comprehensive assessment reveals that the database generated in this study is broadly representative of the CNS of adult locust, providing comprehensive gene information at the transcriptional level that could facilitate research of the locust CNS, including various physiological aspects and pesticide target finding.

## Introduction

The insect nervous system consists of a central nervous system (CNS) and a peripheral nervous system. The CNS is formed by ventral segmental ganglia and the brain, and usually controls the reproduction, metamorphosis, growth, metabolism, and behaviors of insects directly. Insects have provided important model systems for the analysis of the neural networks underlying all kinds of behavior. Diptera such as *Drosophila melanogaster*, have long been used for the study of the nervous system. The nervous system of Orthoptera, which includes the locust, shares many properties with the Diptera. However, large body size makes the locust particularly well suited for investigation into the development (R. Lakes-Harlan 1995), structure, and molecular biology of the nervous system (Ayali et al. 2002; Stern et al. 2007, Braunig 2008). The locust has been a model for developmental processes in the nervous system for decades (Thomas et al. 1984).

The locust phenotypes that are related to its migration and agricultural damage are regulated by the nervous system. The phenotypic phase change makes the locust undergo a transformation between solitarious and gregarious forms, inducing widespread differences in behavior, reproduction, endocrine balance, immunity, physiology, and morphology (Rahman et al. 2003; Kang et al. 2004). It is regulated by substances of the nervous system, such as pheromones, neuropeptides, and neurotransmitters (Rogers et al. 2004; Pener and Simpson 2009), and numerous molecular activities are involved in phase plasticity (Kang et al. 2004; Ma et al. 2006). However, because of the shortage of gene information, the molecular mechanisms of these phenotypes, especially the exact role these nervous system substances play in phase transition, are currently not known. In order to control these worldwide agricultural pests, many pesticides have been designed to disrupt the proteins essential to the normal function of the pest nervous system including voltage-dependent sodium channels (Narahashi et al. 1998; Narahashi et al. 1999; Zlotkin 1999), GABA receptors (Hosie et al. 1997), nicotinic acetylcholine receptors (Massol et al. 2000; Matsuda et al. 2001; Toshima et al. 2009), glutamate-gated chloride channels (Cully et al. 1994), acetylcholinesterase (Zhu and Clark 1995; Zhou and Xia 2009), and octopamine receptors (David and Coulon 1985; Roeder 1999). The study of the locust nervous system may help us to identify efficient insecticide targets, using molecular methods to solve the problems of existing pesticides, such as pollution, low toxicity, and pesticide resistance.

There is no genome sequenced for any locust species at present, in part due to the large size of locust genomes (Pener and Simpson 2009). Although a complimentary project was finished on the CNS of desert locust *Schistocerca gregaria* Forsskåi, with 12,709 EST obtained from two phages (Badisco 2011), research at the molecular level of the locust nervous system is still insufficient. Specifically, the gene information about neuronal transcripts of the migratory locust, *Locusta migratoria manilensis* (L.) (Orthoptera: Acrididae) is scarce, and does not reach research needs. It is necessary for us to develop a complementary project providing more transcript data for functional gene screening and large-scale studies of gene expression in the locust *L. migratoria manilensis* nervous system.

In this study, the Illumina HiSeq™ 2000 platform was used to sequence and analyze the transcriptome of the CNS of *L. migratoria manilensis* in order to find more functional genes related to the locust nervous system, and to provide a useful resource for future study on the reproduction, metamorphosis, growth, metabolism, and behavior of locust.

## Materials and Methods

### Locust culture and total RNA extraction

Adult locusts used in this experiment were obtained from a gregarious population, and cultured according to the description of Gillespie (Gillespie et al. 2000). Locusts were raised with corn sprout food, in 20×20×20 cm cages at 28 ± 2° C, under a 12:12 L:D photoperiod. CNS samples from 80 locusts were harvested according to the description of Rogers (Rogers et al. 2004). Briefly, the whole brain, including the optic lobes, was dissected from the head. The complete thoracic ganglion chain and the ventral nerve cord were also harvested. After harvesting the CNS samples, the total RNA was immediately extracted.

RNA was isolated using Trizol (Invitrogen, USA) according to the manufacturer's instructions. To remove any contaminating genomic DNA from the RNA sample, total RNA was treated with DNase I (Takara Bio, www.takara-bio.com) at 37° C for 30 minutes. Next, total RNA was subjected to the Trizol extraction once again. The RNA quantity and integrity were evaluated by gel electrophoresis, and quantified by measuring absorbance at 260 nm and 280 nm.

### RNA sequencing, data processing and annotation

The total RNA was sent to the Bioinformatics Center of BGI-Shenzhen for RNA sequencing using Illumina/Solexa sequencing technology, as described previously (Cloonan et al. 2008; Mortazavi et al. 2008; Maher et al. 2009; Filichkin et al. 2010).

Transcriptome *de novo* assembly was carried out with short reads assembly program SOAPdenovo (Li et al. 2010). SOAPdenovo first combined reads with a certain length of overlap to form longer fragments called contigs. Then, the reads were mapped back to contigs. With paired-end reads, contigs from the same transcript, as well as the distances between these contigs, were detected. Next, SOAPdenovo connected the contigs, using “N” to represent unknown sequences between two contigs, to generate scaffolds. Paired-end reads were used again for gap filling of scaffolds in order to get sequences with minimal N's and the longest length; these are defined as unigenes.

All of the unigenes were annotated by comparing against nr databases in NCBI and the Swiss-Prot database. Annotation was performed using Blastall software to compare against Cluster of Orthologous Groups (COG) and Kyoto Encyclopedia of Genes and Genomes (KEGG) databases in order to facilitate future studies. Hits with *E-value* < 10^-5^ were considered to be significant matches. If more than one sequence in the existing database had high sequence similarity to a unigene, the most similar one was assigned to the query. The data discussed in this publication have been deposited in NCBI's Gene Expression Omnibus (GenBank ID: GSE24498). The Blast2GO software was used to obtain the Gene Ontology (GO) annotation terms based on the similarity information of gene sequences (Conesa et al. 2005).

### Comparing with the existing locust genes

The unknown unigenes were compared with ESTs of other non-CNS organs, including whole body, head, hind leg, and midgut of *L. migratoria manilensis* in NCBI, with *E* ≤ *1e-05* (Kang et al. 2004). The same genes were filtered out from the dataset in order to get the CNS specific genes.

In order to isolate the specie specific genes, all of the unigenes were compared with the EST database of another locust specie, *S. gregaria* (Badisco 2011). Hits with *E*≤ *1e-05* were considered to be significant matches. The isolated unigenes, including conserved genes and differentially expressed genes, were compared against nr database, and the GO annotation terms were obtained by Blast2GO software.

## Results

### Sequencing and sequence quality

**Figure 1.**
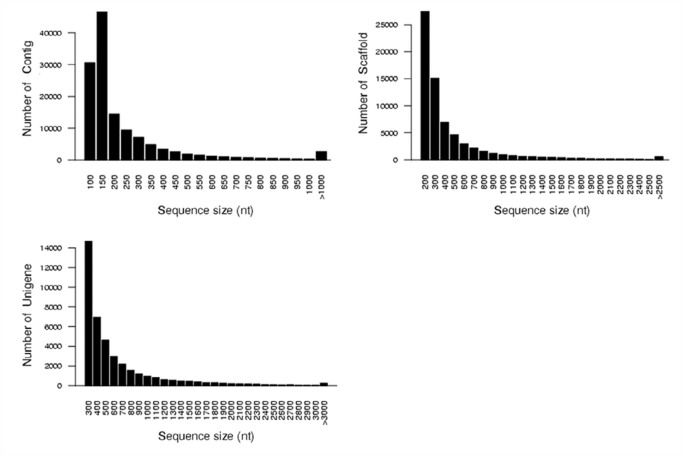
The length distribution of contigs, scaffolds and unigenes. High quality figures are available online.

In this study, 4,276,182,450 total bases from 57,015,766 sequence reads were obtained. Data analysis showed that there were 41,179 unigenes total. A total of 14,611 unigenes were longer than 500 bp, and 5,519 unigenes were longer than 1,000 bp (Table 1). The length distribution of contigs, scaffolds, and unigenes are shown in Figure 1.

### Annotation of the unigenes

**Figure 2.**
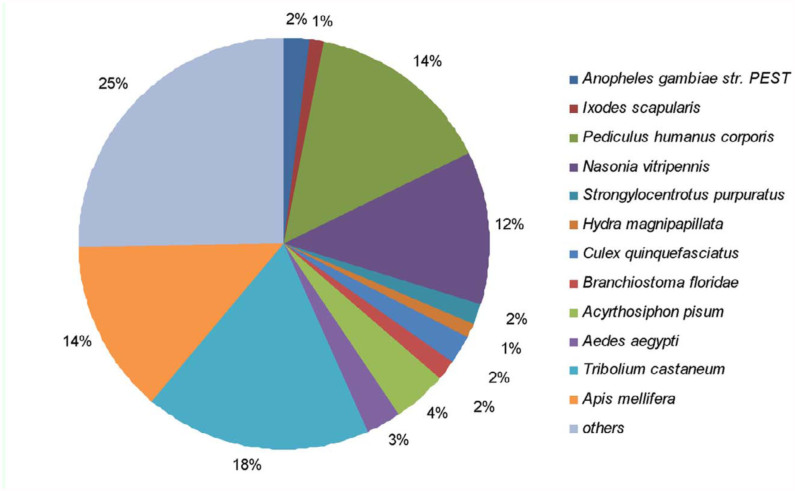
Species distribution of the BLASTX results. This figure shows the species distribution of unigene BLASTX results against the nr protein database (*E*≤ *1e-05*) and the proportions of each species. Different colors represent different species. Species with proportions of more than 1% are shown. High quality figures are available online.

Using BLAST searches of the nr, Swiss-Prot, and KEGG databases, 38.6% of the total unigenes (15,895) were identified (*E*≤ *1e-05*), and the remaining 61.4% (25,284) showed no or low similarity. The species distribution of the best match result for each sequence is shown in Figure 2. The locust CNS showed 18% matches with *Tribolium castaneum* sequences, followed by *Pediculus humanus corporis* (14%), *Apis mellifera* (14%), and *Nasonia vitripennis* (12%).

In the 15,895 identified unigenes, 644 nervous system relevant genes, coding approximately 130 different nervous system genes, were identified based on previous research in insects. The most common nervous system relevant genes were classified into five groups: neural components genes, ion channel related genes, neurotransmitter transporter and receptor related genes, period circadian protein genes, and others. Due to the large number of nervous system relevant genes, only one representative unigene was listed in Table 2 for each typical nervous gene. To estimate the coverage of the CNS genes in this database, the signal transduction component genes, such as neurotransmitter, modulator, transporter and receptor genes, were counted to calculate the coverage (Table 3). The result showed that this database covered most of signal transduction component genes.

**Figure 3.**
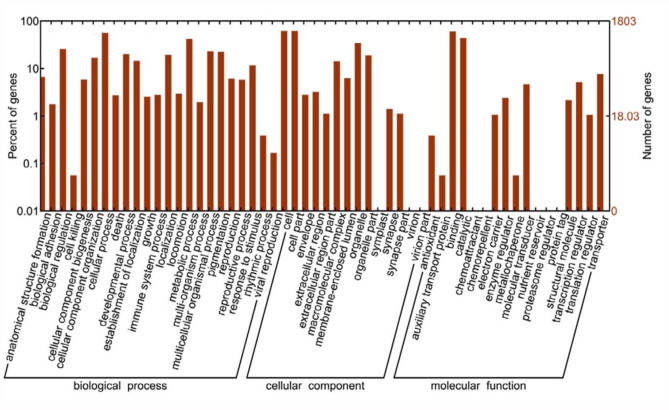
Assignment of Gene Ontology (GO) categories of unigenes. The results are summarized in three main categories: biological process, cellular component, and molecular function. The right y-axis indicates the number of genes in a category. The left y-axis indicates the percentage of a specific category of genes in that main category. High quality figures are available online.

**Figure 4.**
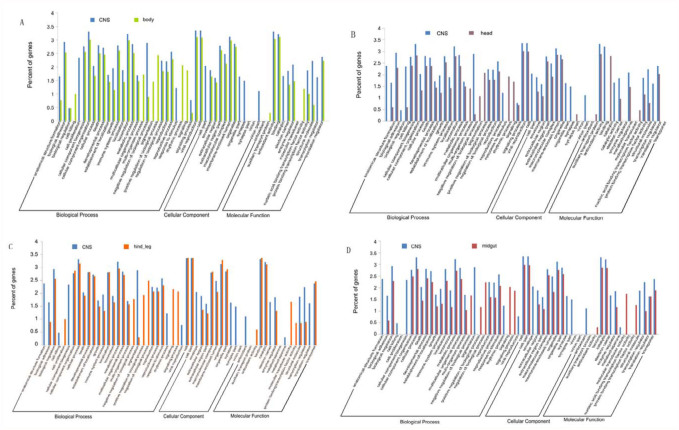
Comparison of CNS unigenes with non-CNS ESTs in NCBI by GO categories. According to the GO functional annotation, the unigenes from locust whole body (A), head (B), hind leg (C) and midgut (D) CNS were compared. The y-axis indicates the logarithm of number of genes in a category. High quality figures are available online.

**Figure 5.**
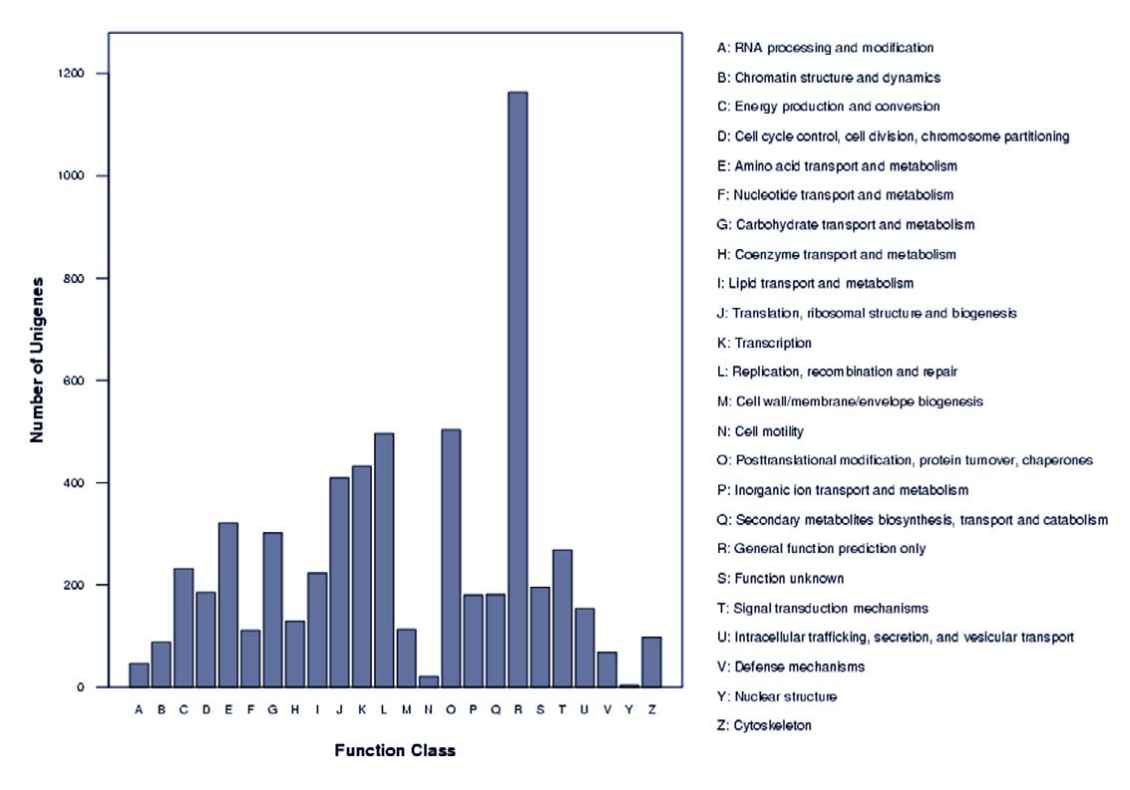
Histogram presentation of clusters of orthologous groups (COG) classification. Among the 25 COG categories, the cluster for “General function prediction” represents the largest group, followed by “Post-translational modification, protein turnover, chaperones” and “Replication, recombination, and repair”. High quality figures are available online.

The 15,895 identified unigenes were then classified by ontology in order to get the GO functional annotation describing the molecular functions, cellular components, and biological processes of unigenes. The unigenes of the locust CNS were classified into 54 major ontology sub-categories (Figure 3). The molecular function ontology reveals that the largest functional categories for CNS transcripts were those related to binding proteins, enzymes, and transporters. This predicted result was consistent with the function of genes in the nervous system. The GO functional annotation items were further compared with the existing non-CNS locust database (Figure 4). The results showed that some new items were generated, which could contain many genes related to the function of the CNS. The annotated sequences were searched for genes involved in COG classification in order to further evaluate the completeness of the library, and the effectiveness of the annotation process. As a result, out of 15,895 nr hits, 5,922 sequences had a COG classification (Figure 5). Among the 25 COG categories, the cluster for “General function prediction” represents the largest group (1,163, or 19.64%), followed by “Posttranslational modification, protein turnover, chaperones” (503, or 8.49%), and “Replication, recombination and repair” (496, or 8.38%).

**Table 1.**
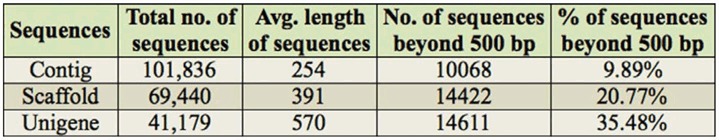
Sequencing results and quality.

**Table 2.**
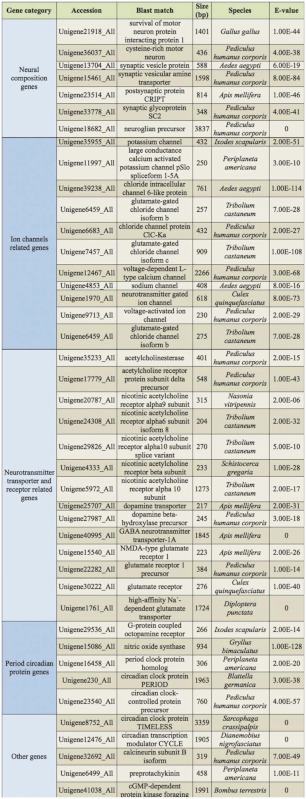
Representative genes of the locust nervous system.

**Table 3.**
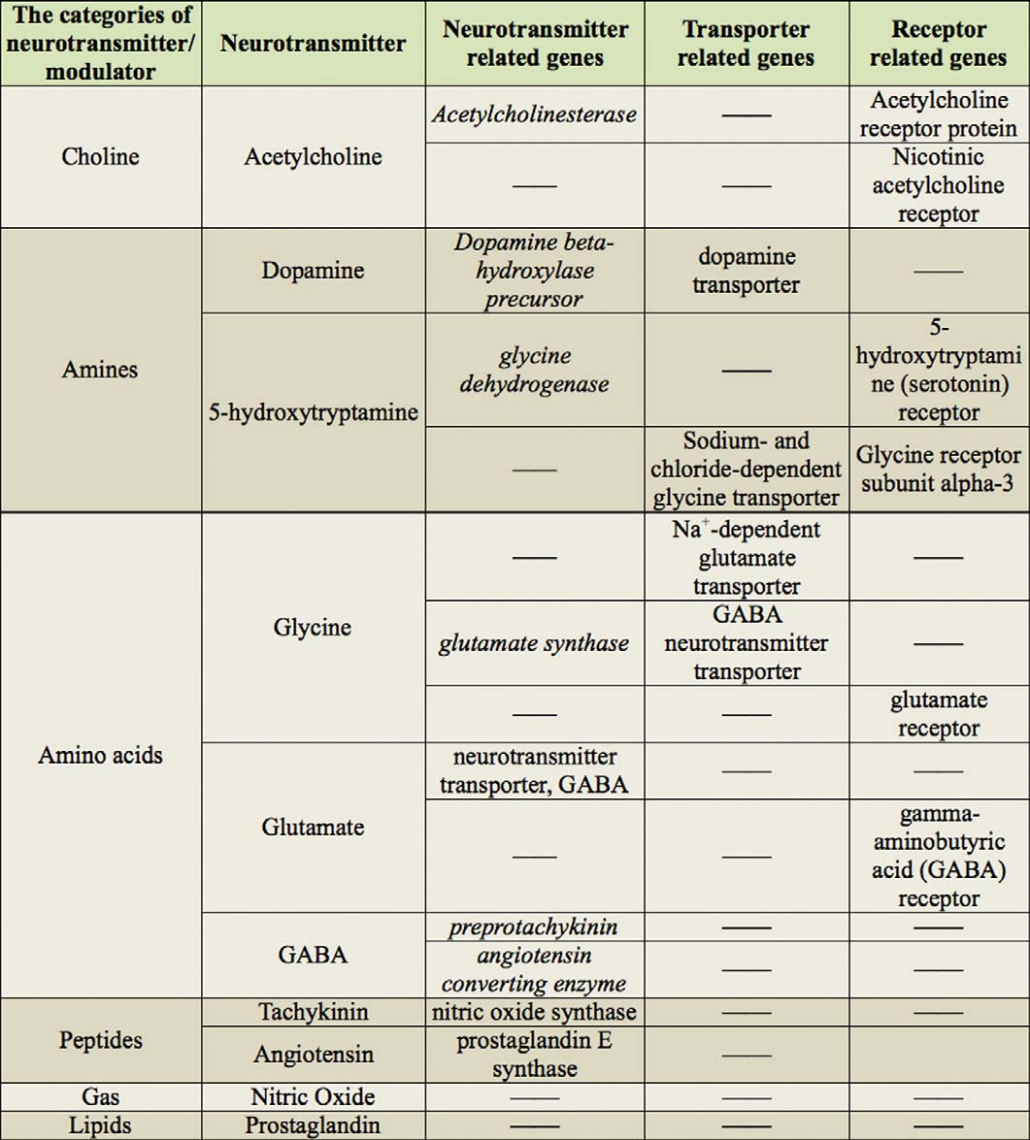
Neurotransmitter, modulator, transporter and receptor related genes.

After comparing with the non-CNS organs in *L. migratoria manilensis*, 62.5% of the unknown genes were similar to previously acquired ESTs from non-CNS organs, and the remaining 9,482 (37.5%) unknown unigenes had no significant similarity to the non-CNS genes. Since the genes from other organs were filtered out, more CNS specific genes could be found in the 9,482 unknown unigenes. However, the other organs included the nerve, which made the CNS share unknown nervous unigenes with other organs. Because of this sharing, some of the unknown nervous genes might also have been filtered.

**Figure 6.**
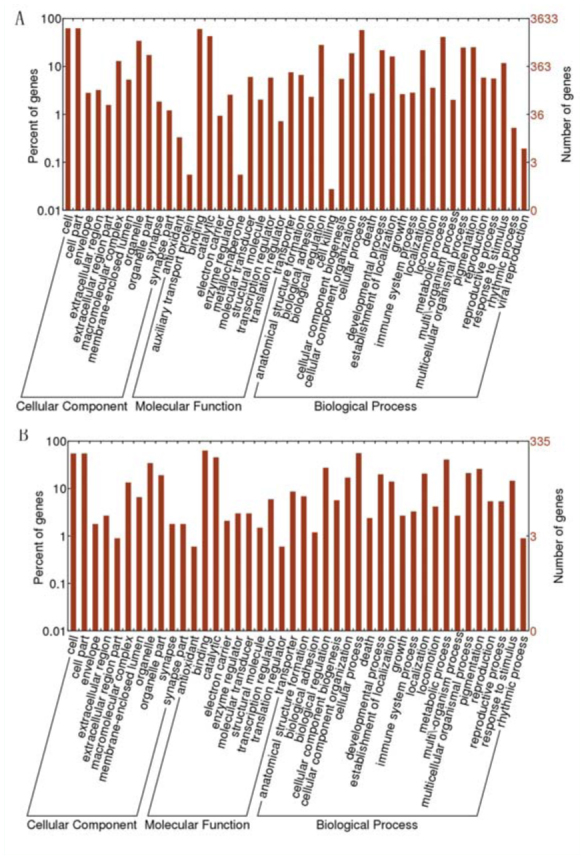
Comparison of CNS unigenes with EST database of *Schistocerca gregaria*. The unigenes in this research was compared with the *S. gregaria* EST database were compared. The differentially expressed genes and conserved genes were annoted by GO functional annotation. Figure 6A shows GO catagories of the differentially expressed genes of *Locusta migratoria manilensi* compared to *S. gregaria*, while Figure 6B shows GO catagories of the conseverd genes. High quality figures are available online.

The unigenes were compared with the *S. gregaria* EST database in NCBI (Badisco 2011). As a result, 32,110 unigenes were differentialy expressed between *S. gregaria* and *L. migratoria manilensis*, while 9,069 unigenes were conserved. There were 3,633 unigenes identified in differentially expressed unigenes, and 335 identified in conserved unigenes. Both the identified unigenes in the differentially expressed and conserved group were classified by ontology in order to get the GO functional annotation (Figure 6). The conserved unigenes were mostly constituted of basic physiological genes, metabolic genes, housekeeping genes, and so on. The differentially expressed genes had three items not found in the conserved genes: viral reproduction, metallochaperone, and auxiliary transport protein.

## Discussion

### CNS genes in the locust

The mean length of assembled transcriptome data (41,179 unigenes) in our study was 570 bp. After annotation, the most common CNS genes were found. GO functional annotation and COG classification showed that the transcriptome library has good coverage, and the annotation was effective. All the results indicated that this database provides a better resource for cloning and study of CNS genes, greatly enriches the current locust database, and will contribute to research with respect to the identification of novel genes, insecticide targets, and various physiological mechanisms.

Using transcriptome sequence analysis, it was surprising to find that *T. castaneum*, belonging to Coleoptera, shared the highest similarity with the locust CNS in the BLAST annotation, whereas *Gryllus veletis*, belonging to Orthoptera, and a fellow locust, showed a very low match percentage (0.17%). These results may be due to the lower availability of sequence resources of Orthoptera. However, even though the numbers of sequences of *T. castaneum*, *P. humanus corporis*, and *Drosophila melanogaster* were comparable, the transcriptome sequence of locust shares only a small number of similar sequences with *D. melanogaster*. This may be due to the phylogenetic relationships between locust and these insects. The phylogenies inferred from both morphological and molecular dataset comparison support that the locusts of Orthoptera had a closer relationship with *T. castaneum* and *P. humanus corporis* than with *D. melanogaster* of Diptera (Andrew 2011; Strausfeld 2011).

The conserved and differentially expressed genes between *S. gregaria* and *L. migratoria manilensi* have been studied. Compared to the large number of differentially expressed genes, the conserved genes played a relatively small part that mainly focused on basic life processes, including the cellular processes, metabolic processes, biological regulation, pigmentation, and so on. The differentially expressed genes shared most of the items with conserved genes. However, some new items were found in the biological processes and molecular function, including viral reproduction, metallochaperone, and auxiliary transport protein. The smaller number of conserved genes suggested that the two locusts had many differences in molecular level, and some of these genes could be due to the different living environments and habits of the two species. These results can help the study of the differences between *L. migratoria manilensi* and *S. gregaria* in behavior characteristics and physiological characteristics.

In this study, many new nervous genes were found. This may be due to the suitability for gene discovery of the new method. Another possibility is that the insects used in this experiment had been raised in cages, which resulted in an environment with a high population density. It is well-known that locusts express different phenotypes in response to local population density, and more nervous genes related to the phenotypes are found in population-dense situations.

### The representative gene of the locust nervous system

Based on previous research, 644 unigenes, coding about 130 different nervous system relevant genes, were identified in this study, including typical CNS genes such as ion channel related genes, neurotransmitter transporter and receptor related genes, and period circadian protein genes.

### Ion channels related genes

The ability of neurons to generate and transmit electrical signals depends on the ion channels that are permeable to ions, including potassium (K^+^), calcium (Ca^2+^), and sodium (Na^+^). Because of their essential roles in signal transduction, the ion channels are the molecular targets for a wide range of drugs, insecticides, and neurotoxins (Bloomquist 1993; Zhao et al. 2005; Nicholson 2007). Due to the high GC content, and the long sequence length, there is a shortage of EST information, and relatively little is known about the properties of ion channel genes in locusts. In this study, 12 genes, including the K^+^ channels, Ca^2+^ channels, and Na^+^ channels, were identified (Table 2), with sequence length between 250 bp and 2,260 bp.

### Neurotransmitters, transporters, and receptors related genes

Neurotransmitters are chemical signals released at nerve terminals. Generally, neurotransmitters are conveyed by transporters, and bind to specific neurotransmitter receptors on the membranes of post-synaptic neurons and transfer the signal (Caveney and Donly 2002). Various neurotransmitters, transporters, and receptors have already been identified in other insects (Osborne 1996; Caveney and Donly 2002). However, the lack of locust gene information made the current molecular methods unapproachable for large-scale application in multi-gene functional studies of the locust nervous system. With the finding of many neurotransmitters related genes in this database, these problems can be easily solved.

Acetylcholine is an important neurotransmitter, and is hydrolyzed by acetylcholinesterase (AChE) after signal transmission. Insect AChEs have attracted interest because of their function in neurotransmission, and are a target of organophosphate and carbamate insecticides. So far, only one AChE gene of the locust has been cloned (Zhou and Xia 2009), and no other AChE ESTs are in the existing databases of locust. Three unique transcripts were found in this study, providing an opportunity for cloning and functional analysis of additional AChEs genes in locust.

Many of the transmitter-related genes were found by sequencing. The neurotransmitters have been shown taking part in locust phase polyphenism (Rogers et al. 2004). However, the exact role these genes play in phase polyphenism is still not clear. With the sequences, the transmitter-related genes can be cloned easily, which could then be used to investigate the phase polyphenism related path-way and regulation mechanism.

### Period circadian protein related genes

The insect period circadian proteins, which are located mainly in the brain, provide organisms with a means of synchronization of life processes and adaptation to environmental cycles. In mammalian, the circadian rhythm can specifically affect the immune system (Lowrey and Takahashi 2004). The same phenomenon has also been well studied in *D. melanogaster* (Nishinokubi et al. 2003; Shirasu-Hiza 2007). Research on *D. melanogaster* circadian genes, including period (per), clock (clk), cycle (cye), shaggy (sgg), and timeless (tim), have provided clues to the mechanisms of the period circadian system (Nishinokubi et al. 2003). The clock genes period and timeless have been demonstrated to be related to photoperiodic sensitivity in *Sarcophaga crassipalpis* (Kostal et al. 2009). The rhythms of locust activities have not been elucidated at the molecular level, and identification of several unigenes of period circadian proteins in this study provide the basis for a detailed analysis of their roles in locust.

### The unknown genes

A total of 61.4% of unigenes had no homologues in existing databases, but they may not be entirely novel genes, since some of the genes in locust may not be highly conserved. Nevertheless, there should be numerous genes with unknown function. The new nervous system relevant unigenes provide a new resource for elaborating on unknown mechanisms in locust nervous system research, as well as in the characterization of signaling pathways. Since the RNA interference (RNAi) technique has been shown to be effective for functional analysis of locust genes (Dong and Friedrich 2005; Zhou and Xia 2009), the functions of the 9,482 genes that were shown to be CNS-specific can be explored by high-throughput RNAi, as the researches did in *Drosophila* (Boutros et al. 2004; Cronin et al. 2009) and *Caenorhabditis elegans* (Fraser et al. 2000; Maeda et al. 2001).

## Abbreviations

CNS,: central nervous system;
nr database,: non-redundant protein sequences database;
**GO**,: gene ontology;
COG,: cluster of orthologous groups;
KEGG databases,: Kyoto Encyclopedia of Genes and Genomes databases;
RNAi,: The RNA interference technique;
NCBI,: National Center for Biotechnology Information;
nt,: nucleotide
